# ERP markers are associated with neurodevelopmental outcomes in 1–5 month old infants in rural Africa and the UK

**DOI:** 10.1016/j.neuroimage.2020.116591

**Published:** 2020-04-15

**Authors:** Laura Katus, Luke Mason, Bosiljka Milosavljevic, Samantha McCann, Maria Rozhko, Sophie E. Moore, Clare E. Elwell, Sarah Lloyd-Fox, Michelle de Haan, the BRIGHT project team, Saikou Drammeh, Ebrima Mbye, Ebou Touray, Mohammed Ceesay, Buba Jobarteh, Momodou K. Darboe, Topun Austin, Andrew Prentice

**Affiliations:** a Centre for Family Research, Department of Psychology, University of Cambridge, UK; b Great Ormond Street Institute of Child Health, University College London, London, UK; c Centre for Brain and Cognitive Development, Birkbeck College, London, UK; d Department of Women and Children's Health, Kings College London, UK; e Department of Medical Physics and Biomedical Engineering, University College London, London, UK; f Medical Research Council, The Gambia at the London School of Hygiene and Tropical Medicine, London, UK; g Department of Psychology, University of Cambridge, UK; h Great Ormond Street Hospital for Children NHS Foundation Trust, London, UK

**Keywords:** Neurodevelopment, Global health, Infants, Event related potentials, Novelty detection, Habituation

## Abstract

**Introduction:**

Infants and children in low- and middle-income countries are frequently exposed to a range of poverty-related risk factors, increasing their likelihood of poor neurodevelopmental outcomes. There is a need for culturally objective markers, which can be used to study infants from birth, thereby enabling early identification and ultimately intervention during a critical time of neurodevelopment.

**Method:**

In this paper, we investigate developmental changes in auditory event related potentials (ERP) associated with habituation and novelty detection in infants between 1 and 5 months living in the United Kingdom and The Gambia, West Africa. Previous research reports that whereas newborns’ ERP responses are increased when presented with stimuli of higher intensity, this sensory driven response decreases over the first few months of life, giving rise to a cognitively driven, novelty-based response. Anthropometric measures were obtained concurrently with the ERP measures at 1 and 5 months of age. Neurodevelopmental outcome was measured using the Mullen Scales of Early Learning (MSEL) at 5 months of age.

**Results:**

The described developmental change was observed in the UK cohort, who exhibited an intensity-based response at 1 month and a novelty-based response at 5 months of age. This change was accompanied by greater habituation to stimulus intensity at 5 compared to 1 month. In the Gambian cohort we did not see a change from an intensity-to a novelty-based response, and no change in habituation to stimulus intensity across the two age points. The degree of change from an intensity towards a novelty-based response was further found to be associated with MSEL scores at 5 months of infant age, whereas infants’ growth between 1 and 5 months was not.

**Discussion:**

Our study highlights the utility of ERP-based markers to study young infants in rural Africa. By implementing a well-established paradigm in a previously understudied population we have demonstrated its use as a culturally objective tool to better understand early learning in diverse settings world-wide. Results offer insight into the neurodevelopmental processes underpinning early neurocognitive development, which may in the future contribute to early identification of infants at heightened risk of adverse neurodevelopmental outcome.

## Highlights

• Infants in low- and middle-income countries are at risk of poor cognitive outcomes.
• There is a need for objective markers of infant brain development across settings.
• We measured infants' event related potentials at 1–5 month in the UK and The Gambia.
• Results show attenuated habituation and novelty responses in the Gambian cohort.
• ERP P3, but not growth measures, were associated with neurodevelopment at 5 months.

## 1 Introduction

Infants and children in low- and middle-income countries (LMICs) are at increased risk of poor neurodevelopmental outcomes. Recent estimates indicate that one third of young children in LMICs do not meet their neurodevelopmental milestones, with potential lifelong effects for individuals and societies (McCoy et al., 2016). The largest proportion of children who do not reach their full cognitive potential live in sub-Saharan Africa (McCoy et al., 2016). Thus far, the risk factors associated with these adverse neurodevelopmental outcomes are poorly understood. However, they are likely to include exposure to multiple and often interrelated poverty-related risk factors, including undernutrition, infections and sub-optimal care (Jensen et al., 2017).

### 1.1 Importance of the early infant period

The first 1000 days of life, spanning the period from conception to around 2 years of age represent a crucial period for neurocognitive development. During this time, foundations for sensory, motor and cognitive development are being lain, making development in this period critical but also exceedingly vulnerable to adverse environmental factors (Andersen, 2003; Rice and Barone, 2000). From conception, the developing brain increasingly specializes in response to the infants' environment (Johnson, 2011). Exposure to environmental adversity during this period may therefore permanently alter developmental trajectories, impacting on later neurocognitive function. Thus far, the impact of early adversity has primarily been studied retrospectively, by examining the long-term neurodevelopmental outcomes of individuals previously exposed to specific risk-factors. Such approaches however are not able to fully capture the complex interplay of prolonged exposure to both risk and resilience associated factors over the course of development, impeding early and targeted intervention. To establish specific pathways between risk-exposure and later neurodevelopmental outcomes it therefore is necessary to longitudinally track both, starting early in development. Whereas single, cross-sectional measurements during early infancy may not be found to predict later outcome, a longitudinal approach provides a more robust indication of infants’ developmental pathways, and thus enable the tracking of consistently atypical response patterns over time (Bussu et al., 2018).

### 1.2 Neuroimaging in global health

The study of infant development in LMICs has been impeded by a lack of methods suitable to study infants across different ages and settings. To date, studies on infant and child development in LMICs have primarily employed behavioral assessment methods. While these can provide important insights into early development in global health settings (i.e. Abubakar et al., 2008), and can be successfully adapted to new settings (i.e. Milosavljevic et al., 2019; Azari et al., 2017; Hamadani et al., 2014; Boivin et al., 2013) they come with several limitations. They are frequently adapted from batteries used in high-income settings and therefore prone to cultural bias - for example due to i) being reliant on infants’ experience with object-play, ii) subjectivity in scoring, leading to inter-rater differences across settings, and iii) taking a long time to administer, therefore limiting their use in routine developmental assessments (i.e. MSEL; Milosavljevic et al., 2019). Neuroimaging has recently gained traction in the study of infant neurodevelopment in global health contexts, offering a means to measure neurodevelopment across cultures, without being reliant on an overt behavioral response by the infant. These methods can be used to measure early correlates of potential later impairments that only manifest behaviorally at a later time, thus providing an indication of which infants are most at-risk and may warrant closer monitoring and early intervention.

Over recent years, the implementation of developmental neuroimaging, specifically functional near infrared spectroscopy (fNIRS, Begus et al., 2016; Lloyd-Fox et al., 2014; Lloyd-Fox et al., 2017, Papademetriou et al., 2014) has been pioneered, and optimized for use in global health research in rural Gambia. Since then the use of fNIRS in global health contexts has widened to research studies in other low-income settings, including Dhaka, Bangladesh (Perdue et al., 2019) and rural India (Wijeakumar et al., 2019), for a review or recent investigations using fNIRS see (Blasi et al., 2019).

The current study forms part of the Brain Imaging for Global Health project (BRIGHT project, www.globalfnirs.org/the-bright-project) which is the first prospective longitudinal project to implement a range of neurocognitive methods in two parallel infant cohorts in the UK and The Gambia, including fNIRS, EEG and eye tracking (for a description of the implementation of these methods in rural Gambia see Katus et al., 2019). Infants are assessed longitudinally at 7–14 days, and at 1, 5, 8, 12, 18 and 24 months of age. In addition to neuroimaging and eye tracking, the protocol of the BRIGHT project includes a range of population specific behavioral measures; MSEL, measures of language development (Communicative Development Inventory [CDI], Language environment analysis [LENA]), and family caregiving assessments (parent-infant interaction videos, questionnaire-based measures). These neurodevelopmental data are collected concurrently with biological, socioeconomic, parental health, nutritional and anthropometric data.

This paper will describe the EEG studies conducted at one and five months of age in the UK and The Gambia as part of the BRIGHT project. The study was implemented to measure habituation and novelty detection, two crucial processes which undergo rapid developmental changes throughout infancy.

### 1.3 ERP based markers of habituation and novelty detection

The bias to preferentially process and attend to stimuli that have not been encountered before poses a crucial road to learning, facilitating exposure to a larger variety of stimuli and thus driving neurodevelopment. A large body of literature has examined these processes using EEG, specifically event related potentials (ERP's), whereby infants are presented with one standard stimulus, occurring at high probability and either one or two ‘oddball’ stimuli occurring at low probabilities. Paradigms fundamentally rest on the assumption that habituation will occur to repeated stimuli on the one hand, and a discriminatory response to novel stimuli on the other.

The ERP response elicited by auditory novelty and habituation paradigms is well-characterized, as consisting of an early negative deflection at around 100 ms (N1), a prominent positive component at around 300 ms (P3) and a final negative central (Nc) component around 500 ms (Kushnerenko et al., 2007). Across development the morphology has been shown to change, with the early N1 being replaced by a P1/N2 complex, occurring rapidly within the first 200 ms after stimulus onset (Čeponien et al., 2002; Määttä et al., 2005). Further, the latency of the P3 and the Nc have been shown to decrease, as is common for ERP responses and attributed to physiological changes in the brain, such as increased myelination (Cheour et al., 1998; Jing and Benasich, 2006).

In newborns, electrophysiological markers of novelty detection, particularly the P3 component, are highly affected by perceptual stimulus properties, such as stimulus intensity (Kushnerenko et al., 2007, 2013). Over the first months of life, these intensity-based responses decrease, giving rise to a novelty-based response from approximately 2 months of age (Kushnerenko et al., 2013; Otte et al., 2013; Van den Heuvel et al., 2015). By contrasting infants’ ERP responses to either deviants of high acoustic intensity or deviants that differ with regard to their novelty relative to other stimuli, a maturational process has been uncovered, showing that from around 2 months of age intensity-based responses subside, giving rise to the emergence of a preferential response to stimulus novelty (Kushnerenko et al., 2013).

### 1.4 Habituation and novelty detection in LMICs

Studying children in rural Kenya between 4 and 12 years of age, Kihara and colleagues found attenuated auditory and visual ERP novelty responses in cohorts affected by malaria (Kihara et al., 2010b), and meningitis (Kihara et al., 2012), compared to typically developing children (Kihara et al., 2010a). These studies provide a first indication that ERP markers of novelty detection may be modulated by exposure to certain types of poverty-related risk. Auditory stimuli such as the ones employed by Kihara et al. (2010b) and Kushnerenko et al. (2007) are relatively unspecific with regard to culture and can be presented without requiring infants’ overt attention to a screen or their engagement with an experimenter. The rapid pace at which stimuli can be presented (approximately one per second) allows quick acquisition of data with a good signal to noise ratio. The ERP paradigm we employed has been used in many contexts and age groups, making it easier to interpret results from a previously understudied population.

Within the BRIGHT cohort, we have recently demonstrated differential developmental patterns of habituation and novelty detection haemodynamic responses between the UK and Gambian cohort using fNIRS (Lloyd-Fox et al., 2019). In temporal regions of the cortex, habituation to repeated stimuli was reduced and activation in response to a novel stimulus was absent in the Gambian cohort at five to eight months of age. In the current paper, we leverage the high temporal resolution of EEG to examine the rapid neural responses of habituation and novelty detection in the same infants at an earlier time window between one and five months of age. As our previous fNIRS investigation (Lloyd-Fox et al., 2019) has not yet examined associations of the observed habituation and novelty brain responses with factors that might indicate higher exposure to risk, such as infant's growth, or with neurocognitive outcome, we aim to investigate these associations in the present study.

### 1.5 Nutritional status and neurocognitive development

Undernutrition during infancy and childhood has been associated with changes in brain structure and function, leading to suboptimal neurodevelopmental outcomes (Georgieff, 2007, Nurliyana et al., 2016, Prado and Dewey, 2014). Hereby, deficiencies in certain micronutrients pre- or postnatally can be associated with lower performance on neurocognitive assessments (Nyaradi et al., 2013). A large body of literature further indicates a relationship between physical growth (a proxy for nutritional status) and neurodevelopment (Black et al., 2017). A recent meta-analysis of infant growth and its association with developmental outcome across different LMICs showed that each unit increase in length in infants under 24 months was associated with higher scores on neurodevelopmental assessment at age 5–11 years (Sudfeld et al., 2015).

### 1.6 Aims and hypotheses

The current study aims to establish the utility of ERP-based markers in infants between 1 and 5 months of age to assess early neurodevelopmental changes in habituation and novelty detection in two parallel study cohorts in the UK and The Gambia. We further aim to examine ERP responses at 1 and 5 months and determine whether these are associated with infants' concurrent neurodevelopmental outcome as measured by MSEL scores at 5 months of age, as well as infants’ growth trajectories between these age points. Our intention is not to make direct comparisons between the two cohorts, but rather to describe developmental trends for each group before examining whether these changes give an indication about a more universal neurodevelopmental mechanism, whereby early habituation and novelty responses may be associated with behavioral outcome across two diverse infant cohorts.

Implementing the previously described, established paradigm using three stimulus conditions (*Frequent* pure tones, *Infrequent* white noise sounds, *Trial Unique*, novel sounds), we predict the following:

1. It is hypothesized that a developmental change from an intensity-based response towards a novelty-based response will occur between the two age points; at 1 month, *Infrequent* white noise sounds are predicted to elicit larger P3 responses compared to *Frequent* and *Trial Unique* sounds. At 5 months, *Trial Unique* sounds are predicted to elicit larger P3 responses than *Frequent* and *Infrequent* white noise stimuli.
2. The above change in novelty detection is predicted to be accompanied by increased habituation to *Infrequent* white noise sounds at 5 months compared to 1 month. No habituation is predicted to occur to *Trial Unique* sounds.
3. It is hypothesized that Gambian infants' habituation and novelty detection responses will show an attenuated developmental shift relative to previously published research.
4. We hypothesize that the developmental change in P3 amplitude will be associated with neurodevelopmental outcome as indicated by the MSEL at 5 months of age, and that the P3 change will account for more variance in neurodevelopmental outcome scores than anthropometric growth measures alone.

## 2 Methods

### 2.1 Participants

**Fig. 1:**
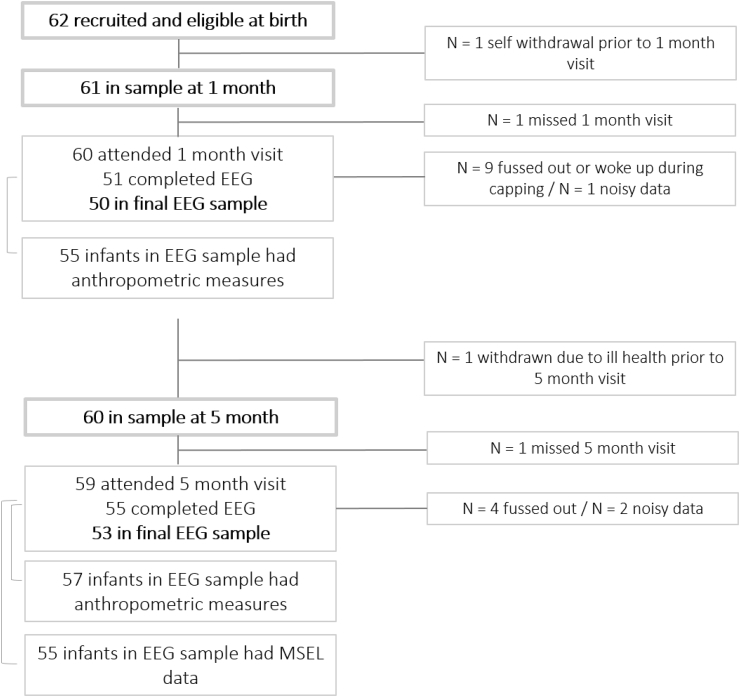
Rates of data retention at 1 and 5 month age points in UK cohort.

Two participant cohorts were assessed, one in the UK and one in The Gambia. For the UK cohort families were approached during their antenatal clinic visit at 32–36 weeks' gestation at the Rosie Hospital, Cambridge University Hospitals NHS Foundation Trust. Families were approached by a member of the research team and given information about the project. Parents expressing an interest were recruited into the study during a subsequent home visit, with informed consent being obtained from both parents. In total, 62 families were recruited into the UK cohort. The majority of families lived either in the university town or in surrounding urban or rural communities within a 20-mile radius. The study was approved by the NHS Health Research Authority (project title ‘Developing brain function for age curves from birth using novel biomarkers of neurocognitive function.’, reference 15/EE/0202, project 178682). Numbers of data included in analyses for the UK sample as well as reason for exclusion are provided in Fig. 1.

**Fig. 2:**
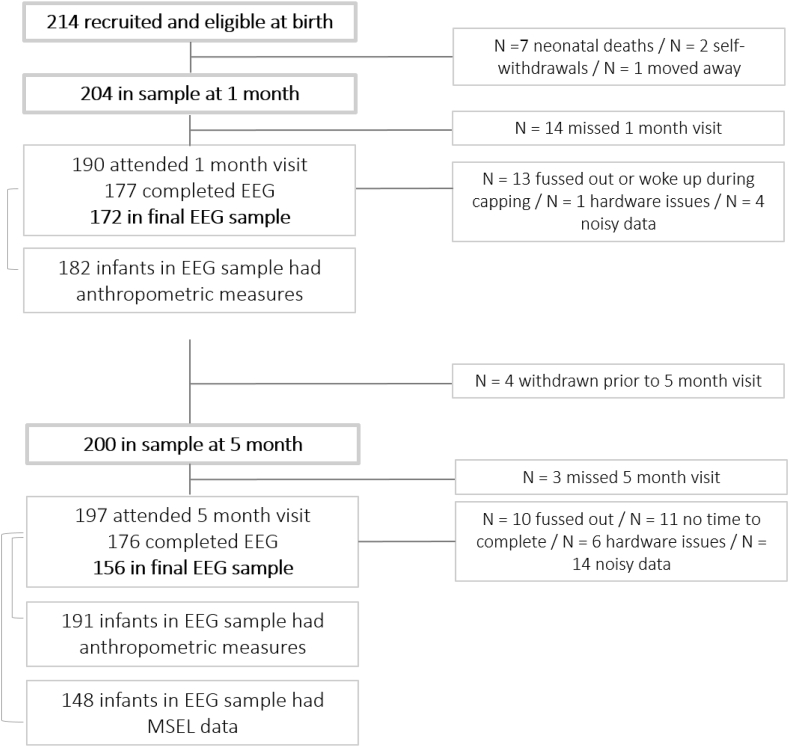
Rates of data retention at 1 and 5 month age points in Gambian cohort.

For the Gambian cohort families were recruited during an antenatal clinic visit to the Medical Research Council Gambia Unit at the London School of Hygiene and Tropical Medicine (MRCG at LSHTM, www.mrc.gm; www.ing.mrc.ac.uk). Families expressing an interest to participate gave informed consent during a home visit. For this cohort, only members of the Mandinka ethnic group were enrolled to avoid confounds of translating some of the language-reliant assessments of the BRIGHT project into multiple languages. While there are multiple ethnic groups in The Gambia, the Mandinka represent the ethnic majority in the West Kiang region (Hennig et al., 2015). In total 214 families were recruited into the Gambian cohort and eligible at birth, who were all residents of either the village of Keneba or surrounding villages in the rural West Kiang District in The Gambia. Numbers of data included in analyses for the Gambian sample as well as reason for exclusion are provided in Fig. 2. Ethical approval was given by the joint Gambia Government – MRC Unit The Gambia Ethics Committee (project title ‘Developing brain function for age curves from birth using novel biomarkers of neurocognitive function’, SCC number 1451v2).

At both sites, infants were included if born between 37 and 42 weeks gestation, and not diagnosed with any neurological deficits during postnatal checks. In the UK cohort, only infants with normal birthweight (>2.5 kg) were enrolled.

The EEG habituation/novelty detection study was performed as part of the 1-, 5-, and 18- month protocols of the BRIGHT project. Here, data from the 1- and 5-month age point are presented. For a proportion of infants, data were missing for one of the following reasons; 1) infants did not allow placement of the cap, or were too fussy to record sufficient data, 2) data were found to be too noisy, with <15 valid trials remaining per experimental condition, 3) infants missing the visit. Numbers of infants retained for the final sample are displayed for Cohort UK and Cohort Gambia in Fig. 1, Fig. 2, respectively alongside details of those infants who contributed anthropometric and MSEL data. For the anthropometric measures, data were missing due to 1) infant excessive fussiness to measurement or 2) lack of time for completion during testing day. For the MSEL, data were missing due to 1) infant non-compliance with items of one or several subscales, preventing the calculation of a composite score, 2) lack of time to complete assessment on the testing day, 3) examiner error preventing scoring for one or several of the subscales.

### 2.2 EEG study

#### 2.2.1 Stimuli and design

**Fig. 3:**
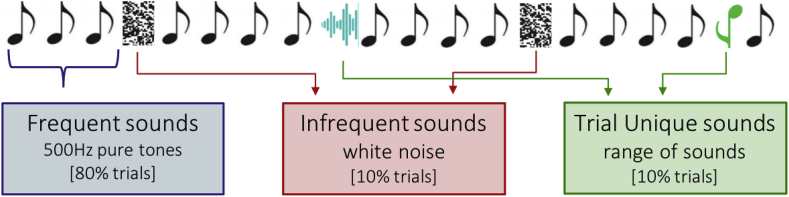
Schematic of stimulus presentation. Presented were three categories of stimuli: *Frequent* sounds consisting of 500 Hz pure tones were presented at 80% probability, *Infrequent* sounds, consisting of white noise segments presented at 10% probability and *Trial Unique* sounds also presented at 10% probability and consisting of clicks, pure tones of different frequencies or digitized vocalizations. *Trial Unique* sounds were different at each presentation and each only presented once during a session.

Infants were presented with auditory stimuli of three different categories (*Frequent/Infrequent/Trial Unique*). *Frequent* sounds consisted of 500 Hz pure tones, presented for 80% of trials, *Infrequent* sounds consisted of white noise segments presented for 10% of trials and *Trial Unique* sounds, also presented for 10% of trials. The *Trial Unique* sounds were each only presented once and consisted of a range of different sounds, such as clicks, tones, digitized vocalizations and syllables (adapted from Kushnerenko et al., 2007). Each sound was presented for 100 ms with a 5 ms ramp up and down period and an inter-stimulus interval of mean length 700 ms, with durations jittered between 650 and 750 ms. Sounds were presented through wireless Sony TMR-RF810R headphones, at a fixed sound level of 60 dB SPL. In total, 1000 trials were presented, with 800 *Frequent*, 100 *Infrequent* and 100 *Trial Unique* trials. The stimulus presentation is displayed in Fig. 3.

#### 2.2.2 Apparatus and procedure

**Fig. 4:**
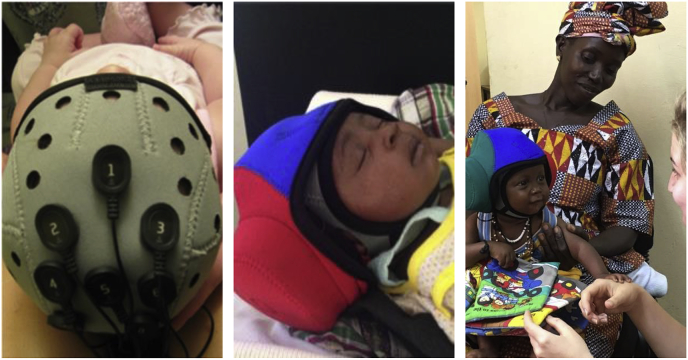
BRIGHT EEG testing set up. Shown are the electrode montage during data acquisition (left) and the testing set up at the 1-month (middle) and 5-month age point (right).

Data were collected using a wireless Neurolectrics Enobio8 system (with low-pass hardware filter at 125 Hz), recording from eight electrodes placed at locations Fz, FC1/2, C1/z/2 and CP1/2 at a sampling rate of 500 Hz. The reference and ground electrode were placed unilaterally on infants' left mastoids and data were recorded in reference to this position. Due to the low density of our recordings, we retained the mastoid reference throughout analysis and did not re-reference to the average of all electrodes. Electrodes were held in place by a neoprene cap which was aligned with anatomical landmarks of infants’ heads. Infants wore a second cap holding in place headphones through which stimuli were presented (Fig. 4).

For the 1-month studies, EEG data were collected while infants were asleep and being held by one of the researchers. For the 5-month studies, infants were assessed while awake with the infant sitting on their parents’ laps while the researchers silently interacted with the infant using quiet toys, bubbles or gesture games. All 1 month old infants were tested while they were asleep. A subset of Gambian infants was also tested asleep at 5 months (*n* = 10) and their data were not included in group analyses for this age point. For both age points, sessions were video recorded to allow for identification of long periods of movement or fussing.

#### 2.2.3 Data pre-processing

Data were pre-processed via automated analysis routines in Matlab 2015b) (Mathworks, Inc, 2015). Data were bandpass filtered offline between 0.5 Hz and 30 Hz, (blackman FIR, with zero-phase, filter order 5500). A high-pass order filter of 0.5 Hz was used to correct for increased slow drift sweat artefact. Data were offset corrected for a timing delay (*X̄* = 32.0 ms, *SD* = 2.6 ms) and segmented from 200 ms pre-to 800 ms post-stimulus onset. Artefacts were rejected via an absolute threshold of >200 μV from minimum to maximum point in each epoch. Similarly, flatlining data with a change of < .1 μV were discarded. To compensate for the large discrepancy of number of presented trials across the three conditions, trial numbers were equalized by identifying the condition with fewest valid trials and taking a random sample of the same size from both other conditions. Datasets with fewer than 15 trials per condition were discarded from further analysis. At the 1-month age point an average of *X̄* = 61.36, *SD* = 16.71 epochs per condition were retained in the UK cohort and *X̄* = 61.95, *SD* = 13.55 in the Gambian cohort. At the 5-month age point an average of *X̄* = 52.72, *SD* = 19.84 epochs per condition were retained in the UK cohort and *X̄* = 52.58, *SD* = 24.82 in the Gambian cohort.

#### 2.2.4 Definition of components

All components were analyzed at electrode Fz in terms of their mean amplitude in the time windows displayed in Table 1, to reduce susceptibility to noise. As infant EEG data tends to be noisier than adult data, it is advised to analyses mean rather than peak amplitude changes, as averaging over multiple sampling points reduces the effect of spurious fluctuations on the average measures (Hoehl and Wahl, 2012). Time windows were defined based on prior literature (i.e. Kushnerenko et al., 2007; Otte et al., 2013, Van den Heuvel et al., 2015) as well as through a cohort-blind inspection of individual waveforms, with averaged time windows capturing the majority of peaks at each age point. To address developmental changes in the ERP morphology, time windows were defined differently at the two age points. As can be seen in Table 1, mean amplitudes of the P3 and the Nc were examined over earlier time windows at 5- than at 1- month, to address known decreases in these components’ peak latencies. Early sensory components undergo significant morphological changes over the first months of life, with a unitary negative component in the newborn period (Kushnerenko et al., 2007; Otte et al., 2013), which develops into a multi-competent complex over the first months and years of life (Kihara et al., 2010b, Kihara et al., 2010a).

**Table 1:** Time windows for mean amplitudes of ERP components per age point.

	Early components			Late latency components	
	N1	P1	N2	P3	Nc
1 month	50–150 ms			250–450 ms	550–750
5 month		60–80 ms	90–110 ms	200–400 ms	500–700

All results presented here are reported from electrode Fz, which has been shown to be principal for novelty responses (Polich, 2007). While novelty responses and particularly the P3 can be measured at different electrode sites, the low density recordings performed in this study prevented such an analysis. Results presented here therefore only reflect the frontal P3 response.

In the current study, the early components were thus examined separately per age point, whereas the P3 and Nc were modelled and examined in terms of their developmental change across the two age points. For estimates of the components’ latencies, peaks were identified for each component within the corresponding time window displayed in Table 2. To increase robustness of this estimate, a jackknife approach proposed by Ulrich and Miller (2001) was applied. We obtained n averages of size n-1, leaving out one of the subjects at each iteration, increasing the signal to noise ratio and the precision of the resulting estimates. Peak latencies of each average were then modelled as individual data points with resulting *F*- and *p*-values corrected as described in Ulrich and Miller (2001), to adjust results for artificial decreases error variance.

**Table 2:** Participant anthropometric growth scores and neurodevelopmental outcome (MSEL scores) for infants included and excluded in final analyses.

	Cohort UK				Cohort Gambia			
	1 month		5 month		1 month		5 month	
	Included	Excluded	Included	Excluded	Included	Excluded	Included	Excluded
Characteristics								
Sex (m/f)	23/27	6/3	27/26	2/3	114/107	28/30	100/110	43/27
	**X ± SD**	**X ± SD**	**X ± SD**	**X ± SD**	**X ± SD**	**X ± SD**	**X ± SD**	**X ± SD**
Age (days)	33.18 ± 5.805	35.44 ± 6.187	155.85 ± 6.663	155.20 ± 5.586	35.24 ± 5.71	35.88 ± 5.66	158.7 ± 8.972	160.4 ± 12.07
Weight (kg)	4.41 ± .538	4.13 ± .461	7.19 ± .898	6.68 ± .829	4.28 ± .573	4.18 ± .602	6.953 ± .840	6.663 ± .074
Length (cm)	53.93 ± 2.507	53.34 ± 1.932	64.53 ± 2.256	63.68 ± 1.613	53.37 ± 2.154	53.12 ± 2.244	64.23 ± 2.293	63.98 ± 1.733
Head circumference (cm)	37.88 ± 1.196	37.48 ± .767	43.04 ± 1.146	42.34 ± 1.829	36.9 ± 1.13	36.85 ± 1.138	41.70 ± 1.51	41.52 ± 1.352
Anthropometric z-scores								
Weight-for-Age	-.066 ± .830	-.771 ± .891	-.136 ± 1.036	-.535 ± 1.158	-.603 ± .992	-.434 ± .093	-.447 ± −1.011	-.9176 ± 1.014
Length-for-Age	-.305 ± 1.209	-.851 ± 1.016	−0.30 ± 1.031	-.600 ± .768	-.852 ± 1.063	-.7436 ± 1.040	-.492 ± 1.019	-.755 ± .838
Head circumference for Age	.698 ± .819	.099 ± .715	.746 ± .839	.298 ± 1.084	-.285 ± 1.034	-.305 ± .941	-.356 ± 1.10	-.65 ± 1.095
Weight-for-length	.10 ± .125	.009 ± .735	.147 ± 1.143	-.100 ± .917	.292 ± 1.105	.227 ± .894	-.104 ± 1.078	-.539 ± 1.124
Neurodevelopmental outcome								
MSEL composite			93.74 ± 11.80	94.26 ± 10.26			96.74 ± 12.68	97.67 ± 15.68
MSEL expressive language			47 ± 5.90	46.75 ± 11.47			46.95 ± 8.23	46.92 ± 8.23
MSEL receptive language			41.48 ± 11.65	43.33 ± 4.041			49.48 ± 13.38	50.00 ± 15.32
MSEL visual reception			47.91 ± 8.715	49.67 ± 4.04			51.22 ± 8.473	50.19 ± 9.51
MSEL fine motor			49.06 ± 8.64	48.5 ± 13.53			45.65 ± 9.847	47.29 ± 10.54
MSEL gross motor			50.62 ± 9.493	52.5 ± 9.43			53.24 ± 12.21	54.89 ± 11.95

Note. Mullen Scales of Early Learning (MSEL) only were administered from 5 months of age.

### 2.3 Mullen Scales of Early Learning (MSEL)

At five months of age, infants were tested using the Mullen Scales of Early Learning (MSEL, Mullen, 1995). The MSEL consists of five subscales which assess visual reception, expressive and receptive language, fine and gross motor development. Subscales contribute to a cognitive composite score, giving an indication of global functioning. The MSEL has been adapted for use with 5–24 month olds in rural Gambia during a previous phase of this project (Milosavljevic et al., 2019). Infants were tested in a quiet room. In the case of infant refusal or disinterest in the task, testing was interrupted and continued later when possible. Caregivers were present during all testing sessions and were asked to positively encourage their infants during testing if they were hesitant to interact with the administrator. Each participant was tested by a trained field assistant, and all sessions were video recorded to allow for inter-rater reliability scoring.

### 2.4 Anthropometric measures

Measurements of length, weight and head circumference (HC) were performed on all infants. Measurements were taken by trained fieldworkers using calibrated tools. Length was measured using a Harpenden Infantometer length board (Holtain Ltd) to a precision of 0.1 cm. Weight was obtained using an electronic baby scale (model 336, SECA) to a precision of 0.01 kg. Finally, HC was measured around the maximum circumference of the head (forehead to occiput) using stretch-proof measuring tape (model 201, SECA) to the nearest 0.1 cm. Each measure was taken in triplicate and the mean of the three measures was used in analyses.

Anthropometric measures were converted to age and sex adjusted z-scores that are based on World Health Organization normative growth data (WHO Multicentre Growth Reference Study Group, 2006). Length-for-Age (LAZ), Weight-for-Length (WLZ), Weight-for-Age (WAZ) and Head Circumference (HCZ) z-scores were computed. Children categorized as “stunted” or “wasted” were identified using WHO criteria based on LAZ and WLZ scores, respectively. Severity of stunting/wasting is categorized as -2SD for moderate and -3SD for severe.

### 2.5 Statistical analysis

For the analysis of developmental changes and cohort differences across the described ERP components, each component was modelled by Condition (*Frequent/Infrequent/Trial Unique*) and Cohort (UK/Gambia). Models for the P3 and Nc further included Age as a factor (1 month/5 month).

To assess the impact of infant size and infant growth during the study period, two separate models were considered: Model 1 included infant size at 5 months of age (using infant WAZ and LAZ) as a measure of infants’ current size. Model 2 included the change in z-scores between 1 and 5 months of age for infant WAZ, LAZ, WLZ and HCZ, as a measure of growth trajectories during this time period.

### 2.6 Data and code availability

All analyses described were performed using EEGLAB (Brunner et al., 2013) and ERPLAB (Lopez-Calderon and Luck, 2014) functions, which are freely available. Data presented in this paper will be made available on request subject to a formal data sharing agreement.

## 3 Results

Participant characteristics, including anthropometric measures and MSEL scores are summarised in Table 2. Data are presented separately for those infants included and excluded in the ERP analyses at 1 and 5 months. No differences were observed between infants included and excluded in the final sample in either cohort with regards to sex, age, weight, head circumference and length or any of the MSEL scales. There was a trend towards inclusion of infants of larger size in the final sample, however in both the Gambian and the UK cohort, with higher WAZ, LAZ, HCZ and WLZ scores for the included compared to the excluded participants. None of these differences however were significant (as indicated by paired sample *t*-test with all *p* > .364) after correction for multiple comparisons.

### 3.1 EEG results

**Fig. 5:**
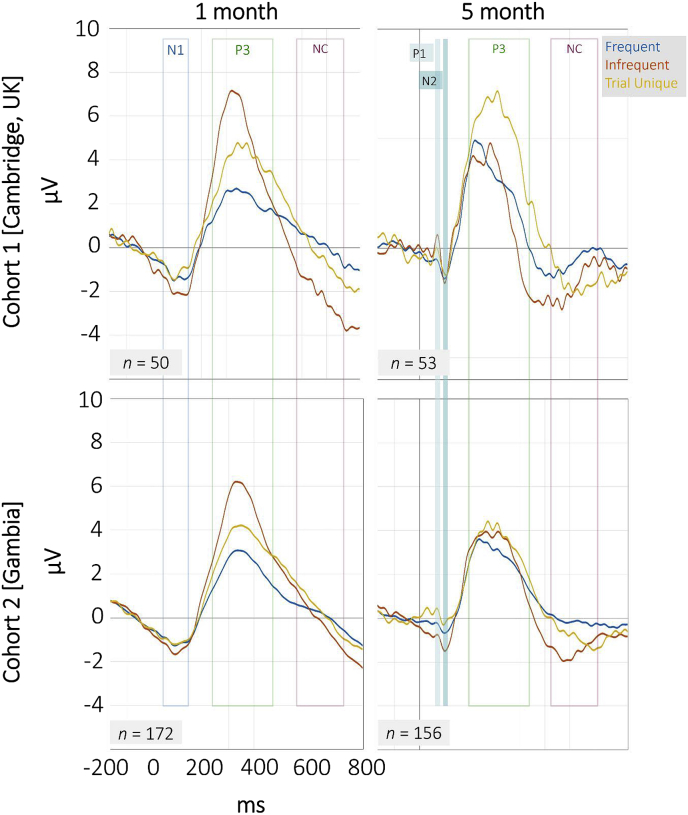
ERP waveforms for the UK cohort (top row) and the Gambian cohort (bottom row) at 1 month (left) and 5 month (right) of age. Displayed are micro voltage changes elicited by *Frequent* (blue), *Infrequent* (red) and *Trial Unique* (yellow) sounds. Time windows over which mean amplitudes for each component were assessed are highlighted.

ERP waveforms per age point and study Cohort can be seen in Fig. 5. Data were modelled using within factors Condition (*Frequent/Infrequent/Trial Unique*) and Age (1 month/5 month) as well as the between factor Cohort (UK cohort/Gambian cohort) in a repeated measures mixed effects ANOVA (RM-ANOVA). Early components (N1 at 1 month, P1 and N2 at 5 months) were modelled by Condition and Cohort only, separately for each age point, whereas the P3 and Nc were modelled using all three predictors.

#### 3.1.1 Early components

For the N1 component measured at the 1 month age point, there were no significant main or interaction effects for factors Condition and Cohort in either the mean amplitudes (0.190 < *p* < .628) or latencies (0.781 < *p*_*corrected*_ < .890). Similarly, at the 5-month age point no significant effects were observed for either the P1 and the N2 components in terms of their amplitudes (P1: 0.134 < *p* < .856, N2: 0.052 < *p* < .242) nor latencies (P1: 0.563 < *p*_*corrected*_ < .746, N2: 0.382 < *p*_*corrected*_ < .691).

#### 3.1.2 P3 component

**Fig. 6:**
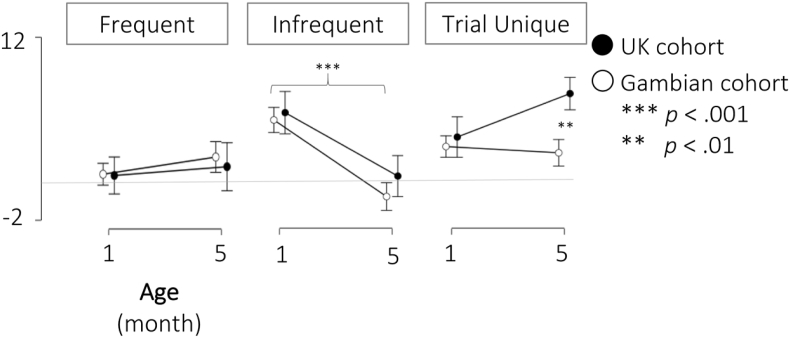
P3 amplitude change between 1 and 5 months of age for *Frequent*, *Infrequent* and *Trial Unique* sounds in the UK cohort UK and the Gambian cohort. Error bars indicate 95% confidence intervals.

Mean amplitudes of the P3 component were modelled in an RM-ANOVA by Condition (*Frequent/Infrequent/Trial Unique*), Age (1 month/5 month) and Cohort (UK cohort/Gambian cohort). Significant main effects were observed for Condition (*F*_*2,334*_ = 31.145, *p* < .001, *n*^*2*^_*p*_ = .157), Age (*F*_*1,167*_ = 7.443, *p* = .007, *n*^*2*^_*p*_ = .043), and Cohort (*F*_*1,167*_ = 6.723, *p* = .010, *n*^*2*^_*p*_ ​= ​.039). Further, significant interactions of Condition * Age (*F*_*2,334*_ = 37.996, *p* < .001, *n*^*2*^_*p*_ ​= ​.185) and Condition * Cohort (*F*_*2,334*_ = 5.635, *p* = .004, *n*^*2*^_*p*_ ​= ​.033) and Condition * Age * Cohort (*F*_*2,334*_ = 3.264, *p* = .039, *n*^*2*^_*p*_ = .019) were observed. This three-way interaction is visualized in Fig. 6. Developmental profiles were found to be similar across the two cohorts for responses to the *Frequent* and *Infrequent* stimuli, with minimal change of the P3 amplitude to the *Frequent* tones across age and uniform decreases with age of the P3 mean amplitude to the *Infrequent* tones in both cohorts. Responses to the *Trial Unique* sounds differed between cohorts, with no change in amplitude between age points in the Gambian cohort (*t*_*122*_ = 0.749, *p* = .455, *d = *0.068) and an increase in the 5 month age point relative to 1 month in the UK cohort (*t*_*45*_ = −3.379, *p* = .002, *d = *−0.498).

Latencies of the P3 were observed to decrease with Age (*F*_*1,167*_ = 9.858, *p*_*corrected*_ = .002, *n*^*2*^_*p*_ ​= ​.056), further there was a significant interaction effect for Age * Cohort, (*F*_*2,334*_ = 4.669, *p*_*corrected*_ = .01, *n*^*2*^_*p*_ = .027), indicating a stronger decrease in peak latencies for the UK compared to the Gambian cohort (UK_1-5_: *t*_*45*_ = 3.21 *p*_*corrected*_ = .002, Gambia_1-5_; *t*_*122*_ = 2.19, *p*_*corrected*_ = .03).

**Fig. 7:**
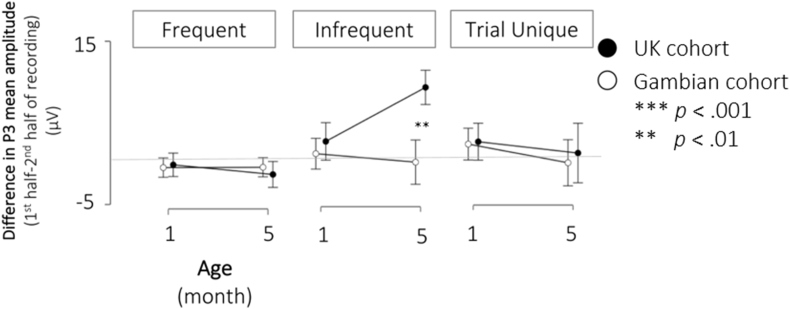
Differences in P3 mean amplitude between first and second half of recording session, per Condition, Age and Cohort. Higher scores indicate a response decrement between the first and the second half of the session. Scores close to 0 indicate no amplitude change over the course of the session.

**Habituation**. To assess the degree of habituation to the three different stimulus categories, we subdivided the recording session and P3 mean amplitudes were averaged for the first and the second half of the session. P3 mean amplitudes obtained during the second half of the recording were subtracted from those measured during the first half of the session. Thus, higher difference scores indicated a larger positive amplitude difference between the beginning and the end of the recording session, reflecting a more pronounced habituation response, whereas lower or negative scores indicated little change or in fact larger P3 responses during the second compared to the first half of the recording session. Difference score was then modelled in an RM-ANOVA by factors Condition (*Frequent/Infrequent/Trial Unique*), Age (1 month/5 month) and Cohort (UK cohort/Gambian cohort). Amplitudes were found to differ significantly by Condition (*F*_*2,298*_ = 7.599, *p* < .001, *n*^*2*^_*p*_ = .049) and Cohort (*F*_*1,149*_ = 5.169, *p* = .024, *n*^*2*^_*p*_ ​= ​.034). Further, interactions were observed between Condition * Age (*F*_*2,298*_ = 3.616, *p* = .028, *n*^*2*^_*p*_ ​= ​.024), Condition * Cohort (*F*_*2,298*_ = 4.971, *p* = .008, *n*^*2*^_*p*_ ​= ​.032) and Condition * Age * Cohort (*F*_*2,298*_ = 3.392, *p* = .035, *n*^*2*^_*p*_ = .022). This latter interaction is displayed in Fig. 7. As can be seen, the effect is driven primarily by a cohort difference in the response to the *Infrequent* tones. Habituation levels were found to be similar at 1 month across cohorts, but greater habituation was observed in the UK cohort at 5 months compared to 1 month (*t*_*45*_ = −4.310, *p* < .001, *d* = −0.650), while levels stayed similar across age points in the Gambian cohort (*t*_*109*_ = 0.0191, *p* = .849, *d* = 0.018). As hypothesized, no habituation effects were shown in response to *Trial Unique* sounds, as evidenced by scores close to 0; further no developmental Age or Cohort difference was observed. Responses to the *Frequent* sounds also showed little habituation, which can be attributed to the number of trials presented for this stimulus category, leading to rapid habituation over the first few trials of the recording session and thus not showing further response decrements over the remainder of the session.

**Influence of state change on P3 response**. As infants were tested during sleep for the 1 month, but awake for the 5 month age point, this state change was confounded with the observed developmental ERP changes. To assess possible differences in the ERP P3 between awake and sleeping infants, we assessed a subset of 5-month-old infants from the Gambian cohort, who were tested asleep (*n* = 10) and whose data did not enter the group analyses described above. These infants were asleep for the majority of the testing day and could thus only be assessed in this state. Data from this subset of infants were compared against a subset of the same size of Gambian 5-month-old infants who were tested awake. The subset of awake infants was chosen randomly, with the exception of being matched on the number of good trials per infant ( ±5 trials compared to infant tested asleep). The effect of sleep on the ERP components was examined by modelling the data in an RM-ANOVA by Condition (*Frequent/Infrequent/Trial Unique*) and State (asleep/awake) to identify differences in P3 mean amplitudes. Age and Cohort could not be entered into this model as only 5-month-old infants from the Gambian cohort entered this analysis. Prior research comparing ERP responses in a sample of sleeping and waking 2-month-old infants (Otte et al., 2013) has reported that while there was an overall effect of state on ERP P3 amplitudes, relative amplitudes across conditions were not affected. It therefore was predicted that a main effect of State would be observed, reflecting general differences in the ERP between sleeping and waking infants, however that Condition responses would be unaffected, indicated by a non-significant Condition * State interaction. In the current analysis however, only a main effect for Condition was observed (*F*_*2,36*_ = 13.998, *p* < .001, *n*^*2*^_*p*_ = .437), but not for State (*F1*_*,36*_ = 0.450, *p* = .641, *n*^*2*^_*p*_ ​= ​.018) or the Condition * State interaction term (*F*_*1,18*_ = 0.132, *p* = .721, *n*^*2*^_*p*_ = .039).

We next examined whether the developmental change between the age points differed for those infants tested asleep at 1 month and awake at 5 months (state change group) and those that were tested asleep at both age points (constant state group). We compared two samples of *n* = 10 infants from the Gambian cohort, using Condition (*Frequent/Infrequent/Trial Unique*) and Age (1 month/5 month) as predictors. As above, Cohort could not be used as a factor, as only infants in the Gambian cohort could be considered. For the constant state group, there was a main effect for Condition (*F*_*2,36*_ = 4.89, *p* = .0132, *n*^*2*^_*p*_ = .214) but only a trend for Age (*F*_*1,18*_ = 4.207, *p* = .055, *n*^*2*^_*p*_ ​= ​.189) and an interaction of Condition * Age (*F*_*2,36*_ = 10.529, *p* < .001, *n*^*2*^_*p*_ = .369). For the state change group, the same pattern of effects emerged with main effects for Condition (*F*_*2,36*_ = 3.89, *p* = .030, *n*^*2*^_*p*_ = .178), but not for Age (*F*_*1,18*_ = 4.081, *p* = .059, *n*^*2*^_*p*_ = .185) and an interaction of the two (*F*_*2,36*_ = 10.586, *p* < .001, *n*^*2*^_*p*_ = .370). While these findings are not to say that EEG measurements are unaffected by participant state, they suggest that in light of this ERP study, the P3 amplitude and therefore the main outcome measure obtained from sleeping and awake infants did not differ significantly which facilitates an interpretation of changes observed between age points in terms of neurodevelopmental changes.

#### 3.1.3 Nc component

For the Nc, significant effects were observed for Age (*F*_*1,167*_ = 4.654, *p* = .032, *n*^*2*^_*p*_ = .027), with larger negative amplitudes at 5 compared to 1 month. No main effects were observed for factors Cohort (*F*_*1,167*_ = 0.0798, *p* = .373, *n*^*2*^_*p*_ < .001) and Condition (*F*_*2,334*_ = 1.46, *p* = .234, *n*^*2*^_*p*_ = .009), further none of the interaction terms indicated significant effects. Nc latencies were found to decrease with Age (*F*_*1,167*_ = 4.241, *p*_*corrected*_ = .041, *n*^*2*^_*p*_ = .019), with none of the other main or interaction effects indicating significant differences.

### 3.2 Associations of P3 change and neurodevelopmental scores

**Fig. 8:**
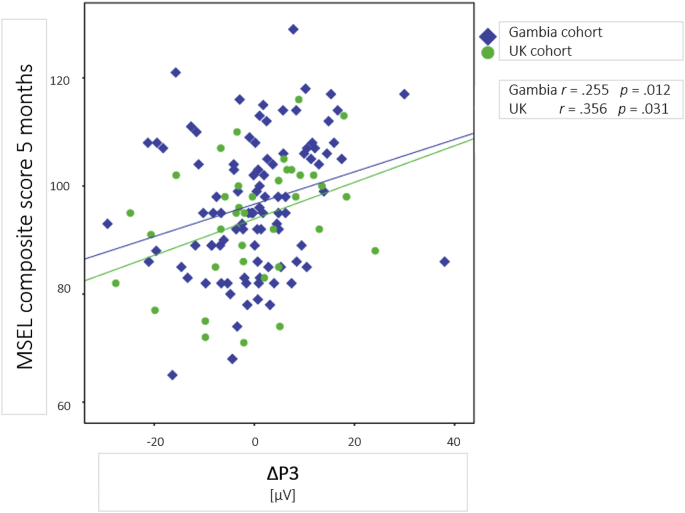
Correlation of ΔP3 and MSEL cognitive composite score at 5 months for the Gambian (blue diamonds) and UK (green circles) cohort. ΔP3 and MSEL scores were significantly correlated in both cohorts with larger ΔP3 scores being associated with higher MSEL composite scores.

Based on the component analysis above, which replicated developmental changes in P3 amplitude over the first months of life, associations between the change in P3 amplitude and neurodevelopmental outcome were examined. To investigate the developmental change in the difference in P3 mean amplitude between the *Infrequent* and *Trial Unique* conditions we calculated a ΔP3 by subtracting the condition difference at 5 months from that measured at 1 month. Fig. 8 shows the association of ΔP3 and MSEL cognitive composite scores for the UK and the Gambian cohort. Significant correlations were observed between ΔP3 and MSEL cognitive composite scores for both the UK (*r* = 0.356, *p* = .031) and the Gambian (*r* = 0.255, *p* = .012). A comparison of the standardized correlation coefficients revealed that there was no difference in the magnitude of the correlation between the UK and the Gambian cohort (*z* = 0.578, *p* = .282).

In two linear regression models, we examined the amount of variance accounted for in MSEL scores at 5 months, by growth indicators and ΔP3. For Model 1, WAZ and LAZ at 5 months were examined as measures of infants’ current size. Predictors were found to be strongly correlated with one another (*r* = 0.592, *p* < .001). To prevent collinearity of predictors in the regression model, each variable was entered into a separate univariate regression models. A weak trend was found between MSEL scores and LAZ at 5 months (*t* = 1.24, *p* = .12). LAZ was subsequently entered as a predictor alongside ΔP3 for Model 1. For Model 2, changes between 1 and 5 months of age in infant WAZ, LAZ, HCZ and WLZ were examined as predictors. Again, predictors were found to be strongly correlated (all r > 0.308, all p < .001). To reduce the number of predictors, each was fit into a univariate model. Only delta change in HCZ showed a trend towards statistical significance (*t* = 1.676, *p* = .095). HCZ and ΔP3 were thus entered into Model 2. To control for type I errors in this exploratory analysis, results for this exploratory analysis were corrected using the False Discovery Rate (FDR) method (Benjamini and Hochberg, 1995).

Neither of the anthropometric indicators in Model 1 (LAZ at 5 months) or Model 2 (delta change in HCZ between 1 and 5 months) were found to be associated with MSEL scores at 5 months (*t*_*Model1*_ = 0.308, *p*_*Model1FDR*_ = 0.828*, t*_*Model2*_ = 1.239, *p*_*Model2FDR*_ = 0.327). In both models, ΔP3 was found to be a significant predictor of MSEL scores at 5 months of age (*t*_*Model1*_ = 3.382, *p*_*Model1*_ <0.001*, t*_*Model2*_ = 3.4, *p*_*Model2*_ <0.001). The null model including only the growth predictors was found to explain little variance in MSEL cognitive composite scores at 5 months of age (*R*^*2*^_*Model1*_ < 0.001, *R*^*2*^_*Model2*_ = 0.010). The R^2^ change when adding the ΔP3 to the model was significant for both models (*R*^*2*^_*Model1*_ = 0.080 *p*_*Model1*_ < 0.001, *R*^*2*^_*Model2*_ = 0.080, *p*_*Model2*_ < 0.001). All regression models can be found in Supplementary Material 1.

To assess whether this association was primarily driven by one of the two cohorts, the above model was fit for each cohort separately. For the UK cohort the anthropometric measures were not found to be associated with MSEL composite scores at 5 months (*t*_*Model1*_ = 0.730, *p*_*Model1FDR*_ = 0.628, *t*_*Model2*_ = 1.430, *p*_*Model2FDR*_ = 0.278), whereas ΔP3 was a significant predictor when added to the model (*t*_*Model1*_ = 2.323, *p*_*Model1FDR*_ = 0.048, *t*_*Model2*_ = 2.501, *p*_*Model2*_ = 0.017). R^2−^change values were found to be of larger magnitude than those reported for the model fit to both cohorts (*R*^*2*^_*Model1*_ = 0.136, *R*^*2*^_*Model2*_ = 0.151). For the Gambian cohort, the same pattern of results emerged, with no associations observed in either model (*t*_*Model1*_ = 0.163, *p*_*Model1FDR*_ = 0.871, *t*_*Model2*_ = 0.456, *p*_*Model2FDR*_ = 0.780), whereas ΔP3 was when added to the model *t*_*Model1*_ = 2.559, *p*_*Model1FDR*_ = 0.039, *t*_*Model2*_ = 2.531, *p*_*Model2FDR*_ = 0.039)*.* R^2^ - change values were found to be similar as for the model fit to both cohorts (*R*^*2*^_*Model1*_ = 0.065, *R*^*2*^_*Model2*_ = 0.064). These results suggest that a significant proportion of variance in MSEL cognitive composite scores is accounted for by ΔP3 scores in both cohorts studied, whereas infant growth in this analysis did not account for a significant proportion of variance at the examined age points. They further suggest that ΔP3 might be better suited to give an indication for neurodevelopmental outcome in a more homogenous sample such as the one studied the UK. Regression models for each cohort can be found in Supplementary Material 2.

## 4 Discussion

The present study successfully implemented ERP measures of auditory habituation and novelty detection to study neurodevelopmental changes in infants in the UK and The Gambia over the first months of life. This provides the first demonstration of the utility of ERP measures in infants during the first year of life in rural Africa. Prior research has suggested a developmental shift over the first months of life, whereby newborns’ electrophysiological response to novelty are driven by perceptual stimulus properties, such as stimulus intensity, whereas the novelty of a presented stimulus becomes the driving factor of these responses over the first months of life (Kushnerenko et al., 2013). This has been interpreted as the emergence of more mature novelty response, during which those stimuli that have not been encountered before are preferentially processed. Our analyses showed that this developmental change towards a greater neural response to stimulus novelty between 1 and 5 months was seen in the UK cohort, but not in the Gambian cohort. Using this previously defined change as a marker of early neurodevelopment, the current study demonstrated that the change in P3 amplitude was found to be associated with concurrent neurocognitive development as measured by MSEL scores at 5 months, and that it accounted for variance in MSEL scores that was not accounted for by anthropometric measures alone. The rate of maturation of the ERP novelty response may thus represent one of the neural correlates underpinning early neurocognitive development. Due to its higher specificity to neurocognitive development than measures such as anthropometric growth it may be able to provide a more robust indicator of infant neurodevelopmental progression.

### 4.1 UK cohort

In line with the hypotheses and prior literature, 1-month-old infants’ P3 response was driven primarily by stimulus intensity, rather than novelty, as evidenced by larger amplitudes to the infrequently presented white noise sounds, compared to both other conditions. The expected change to preferential processing of the novel *Trial Unique* sounds at 5 months was also observed in the UK cohort, with *Frequent* and *Infrequent* stimuli eliciting P3 responses of similar magnitudes, and largest amplitudes being elicited by *Trial Unique* and thus truly novel stimuli. Data from this cohort thus support the development of a novelty response by 5 months of age. It was further hypothesized that the developmental change towards a novelty-based response would be associated with enhanced habituation to the infrequently presented stimuli, despite their sensory intensity. For the UK cohort, the data suggested an enhanced habituation response at the 5- compared to the 1-month age point. Lastly, latencies of the P3 and Nc components decreased with age, which is in line with commonly documented latency decreases with increasing infant age (Jing and Benasich, 2006).

### 4.2 Gambian cohort

At the 1-month age point, response patterns in the Gambian cohort were similar to those described for the UK cohort, whereby larger P3 responses were elicited for the *Infrequent* white noise stimuli than for the other two stimulus categories. However, the developmental shift towards a novelty-based response at 5 months of age was not fully evident in this group. Instead, both *Infrequent* and *Trial Unique* sounds elicited P3 responses of similar amplitudes at 5 months of age, which suggests that on the group level the anticipated response reversal may not have occurred in this cohort by 5 months of age. This notion was further supported by the habituation patterns, which showed no developmental gain in the ability to block out the *Infrequent* white noise stimuli in the Gambian cohort. Regarding other developmental changes, such as the anticipated latency decrease, it was found that latencies were shorter at 5 compared to 1 month of age in the Gambian cohort for both the P3 and the Nc component, however for the P3 component it was also shown that the decrease was less pronounced than that observed in the UK cohort.

Taken together, these findings indicate less pronounced habituation and novelty responses in the Gambian cohort. Particularly the lack of a developmental change towards preferential processing of the novel, trial unique stimuli warrants further investigation, both in terms of associated environmental factors and its associations with later outcome. As no cohort differences were observed in the earlier, sensory-driven ERP components, it is unlikely that these differences can be accounted for by differences in sensory processing alone. A recurrent finding regarding both the novelty as well as the habituation responses was that responses were highly similar at the 1-month age point but grew more disparate towards the 5- month age point. At this point it cannot be determined whether this is reflective of a culminating effect of exposure to environmental risk or progressive adaptation to infants’ current environmental context. Data are currently being collected as part of the 18 month age point of the BRIGHT protocol. Through the inclusion of this further age point, it will be possible to determine whether the disparity represents a temporary diversion in trajectory that resolves towards a later age, or whether trajectories stay separate, representing a qualitative sustained difference in development.

Findings of the current study are in line with recent investigations of the same infant cohorts at 5- and 8- month of age, using an fNIRS task examining habituation and novelty detection (Lloyd-Fox et al., 2019). Using an fNIRS specific paradigm and investigating infants at 5 and 8 months of age, one finding in this fNIRS study was that a developmental change towards more proficient habituation and a stronger novelty response observed in the UK cohort was much reduced in the Gambian infants. The current study extends these findings by showing that these developmental differences are already detectable earlier, during the first 5 months of life. Together these findings suggest some robustness of the observed effects, which can be elicited at multiple age points, using different methods and paradigms. While similar group-level trends are observed on both the fNIRS and this EEG study, a next step will be to interrelate both measures on an individual level to better understand cohort, but also individual differences, in novelty detection, not only in functional terms but also in relation to activation in relevant cortical areas.

### 4.3 Association of ERP-based marker and neurodevelopmental outcome

The current study provides evidence for the potential of the proposed ERP feature to be associated with neurodevelopmental outcome. Using the P3 as a marker, those infants who showed a larger developmental change towards a robust novelty response scored higher on the MSEL cognitive assessment. The P3 change accounted for a significantly larger amount of variance in MSEL scores, than anthropometric growth scores alone. In fact, neither infants’ current size nor growth between 1 and 5 months was found to be associated with MSEL score at 5 months.

While several studies have shown associations between infant growth and neurodevelopmental outcome, this relationship has been proposed to be less robust during the first 6 months of life. A recent investigation of infant growth between birth and 2 years of age across eight LMICs showed an association of growth between 6 and 24 months of age and neurodevelopmental outcome at age 2 (Scharf et al., 2018). However, this study also indicated that growth markers obtained between 0 and 12 months of age were less strongly associated with neurodevelopmental outcome at 24 months than growth markers obtained between 12 and 24 months of age. Neuroimaging markers such as the ones proposed here may thus be more sensitive to identify at-risk infants during this early period.

While instruments such as the MSEL have been able to detect cohort differences from around 12 months of age, group differences are less readily identified during the first months of life (Milosavljevic et al., 2019). The association of the ERP P3 marker with MSEL scores at 5 months demonstrates its potential to detect subtle differences between individuals, before differences become apparent on a group level. It therefore may indicate a potential mechanism underpinning early neurodevelopment. The notion that a reduced bias to orient to novel stimuli may have implications for early learning and thus a broad spectrum of early neurodevelopmental changes has been has long been proposed (Rovee-Collier, 1984). The current study illustrates how these subtle biases can be exploited to inform our understanding of early neurocognitive development across diverse infant cohorts. While neurobehavioral measures such as the MSEL have greatly added to our understanding of infant development in diverse settings around the world, they are less-well suited to inform our understanding of such developmental mechanisms. Until recently, neurobehavioral methods were the only means to assess infants in remote, low-resource settings. The ever-advancing technological improvements in hardware associated with electrophysiological studies such as the one presented here, have now enabled their implementation in the field, through battery-powered, wireless and highly portable equipment.

Assessments of how neurodevelopmental trajectories generalize across settings may be facilitated by a wider implementation of direct measures of neuronal responses, as compared to neurobehavioral measures. First, little adaptation is required across study sites or age groups to implement markers of brain function such as the one described due to their relative objectivity. Secondly, a higher degree of standardization can be achieved across administrators, further facilitating comparisons across sites. In the current study, the P3 change was found to be associated with neurodevelopmental outcome in both the UK and the Gambian cohort, this association was stronger in the UK cohort. While indicating the association might generalize across two vastly different cohorts, this also indicates that electrophysiological marker, such as the one described might be more accurate in relatively homogenous, high-income settings.

In sum, our study demonstrates the potential of understanding early neurodevelopmental mechanisms through the use of ERP markers in infants below six months of age. Recent investigations have demonstrated an association between ERP visually evoked potentials (VEP) and neurodevelopmental outcome in Bangladeshi infant and toddler cohorts (Jensen et al., 2019). In the same cohorts, it could be demonstrated that ERP's elicited in response to faces were attenuated in toddlers (though not infants) experiencing repeated episodes of inflammation. ERP responses were further found to mediate the association between episodes of inflammation and later IQ (Xie et al., 2019, elsewhere in this issue). In line with these studies, our findings highlight the potential for electrophysiological markers to inform our understanding of the neural correlates and neurodevelopmental sequela of early adversity. Furthermore, such measures might be able to offer insights earlier than behavioral assessment methods and could thus play a crucial role in early identification of at risk infants.

### 4.4 Limitations

The underlying neural structures of the novelty response and particularly the P3 encompass a range of cortical and subcortical structures and measuring responses concurrently at different electrode sites enables additional conclusions to be drawn on the underlying neural network differentially giving rise to responses at different sites. Our study is limited in that such conclusions could not be drawn from a unitary focus on the Fz electrode. In this first study, we focused on a smaller electrode cluster in order to increase data quality, however future investigations should consider higher density recordings, which hold potential to enable additional conclusions on the neural origin of the measured signal.

We further found that the hot and humid climate in The Gambia led to increased artefact, specifically to increases in slow drift associated with increased sweating. While this was addressed by implementing a higher high pass filter during pre-processing, it needs to be acknowledged that this issue might affect the comparability of our findings with previously published research.

Another limitation of this study is that data were acquired in different states (asleep at 1 month/awake at 5 months) which complicates conclusions regarding the developmental change. As discussed above, previous studies implementing this paradigm have found differences in the ERP waveform when testing 2-month old infants awake and asleep (Otte et al., 2013), but that state change did not affect the relative differences between conditions. The current study is reliant on examining condition differences at two developmental age points, which therefore should be relatively unaffected. Indeed, in the current study no differences were observed between two subsets of infants who were tested asleep and awake at 5 months of age. While the absence of a state change between experimental age points may be preferable, vast developmental changes during the first months of life necessitate protocols to be adaptive to these changes. Longitudinal studies should therefore employ study designs containing meaningful condition contrasts to make them less affected by state changes.

## 5 Conclusion

We have shown that electrophysiological markers obtained during the first 5 months of life are associated with concurrent neurodevelopmental outcome. The paradigm we chose to replicate offers an objective non-culture specific marker, which can provide an early indication for potential developmental delay in a range of populations. Future research will aim to also assess the medium to long-term predictions of the measures obtained during early infancy and later cognitive development (currently being measured in the BRIGHT study across 12–24 months of age). The current findings suggest a certain degree of universality, given that the association between the proposed marker and concurrent neurodevelopmental outcome was observed across the two different cohorts, yet further research is needed to confirm the utility for the proposed marker in other cohorts and for long-term neurodevelopmental outcomes.

## Funding

This research project is funded by the Bill and Melinda Gates Foundation Grants OPP1061089 and OPP1127625. The Nutrition Theme at MRCG is supported by the MRC & the Department for International Development (DFID) under the MRC/DFID Concordat agreement (MRCProgramme MC-A760-5QX00). This work was further supported by a Child Health Research CIO PhD Studentship and an ESRC Postdoctoral Fellowship, grant number ES/T008644/1 held by the first author. This work is supported by the NIHR GOSH BRC. The views expressed are those of the author(s) and not necessarily those of the NHS, the NIHR or the Department of Health.

## Declaration of competing interest

The authors declare no competing interests.
