# Supplementary material for: ERP markers are associated with neurodevelopmental outcomes in 1–5 month old infants in rural Africa and the UK
Source: Neuroimage. 2020 Apr 15;210:116591. doi: 10.1016/j.neuroimage.2020.116591 (PMC7068721; doi:10.1016/j.neuroimage.2020.116591)

**Supplementary Material**

# Manuscript: Katus et al. ‘ERP markers are associated with neurodevelopmental outcomes in 1-5 month old infants in rural Africa and the UK’.

1. **Linear regression including UK and Gambian Cohort.**

Model 1. Length-for-age z-scores (HAZ) at 5 months and deltaP3 to predict MSEL scores at 5 months


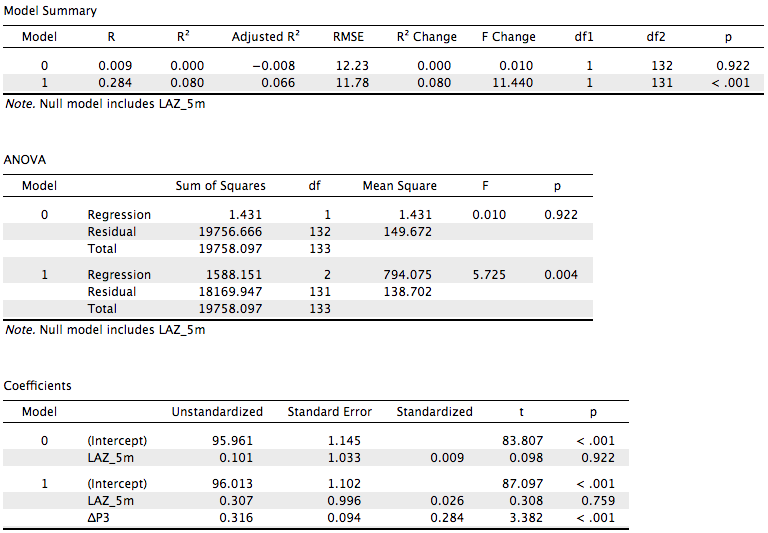


Model 2. Head-circumference-z-score change between 1 and 5 months (HCZchange) and deltaP3 to predict MSEL scores at 5 months
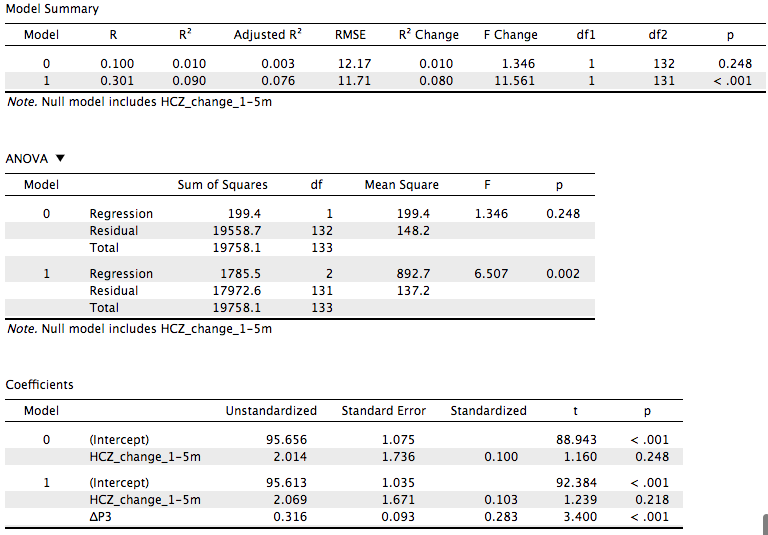


1. **Linear regression for Gambian and UK cohort separately**

**Model 1. Length-for-age z-scores (HAZ) at 5 months and deltaP3 to predict MSEL scores at 5 months. UK only.**

**
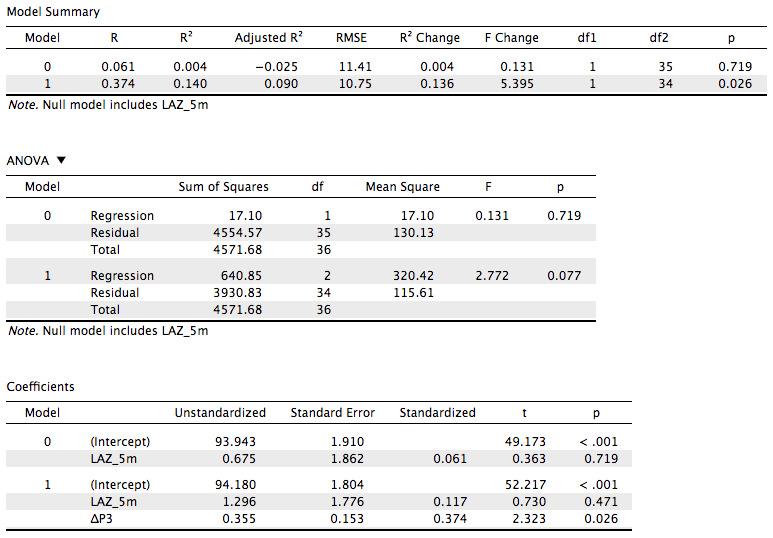
**

**Model 1. Length-for-age z-scores (HAZ) at 5 months and deltaP3 to predict MSEL scores at 5 months. Gambia only.**


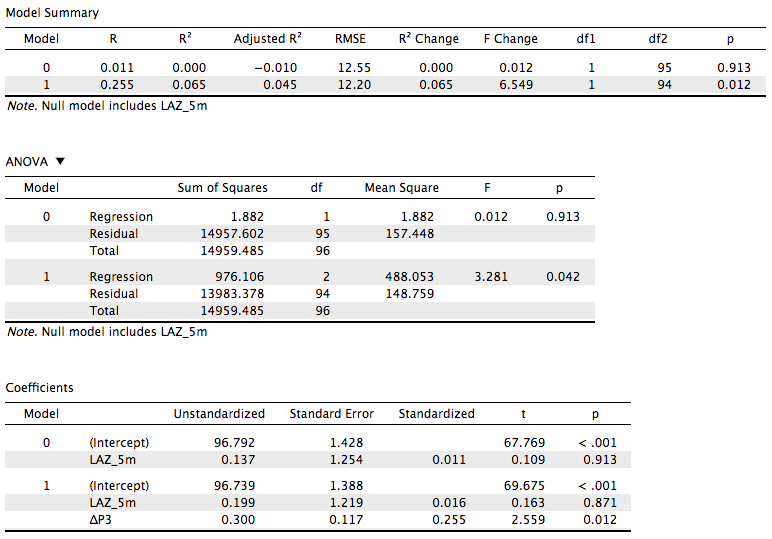


**Model 2. Head-circumference-z-score change between 1 and 5 months (HCZchange) and deltaP3 to predict MSEL scores at 5 months. UK only.**


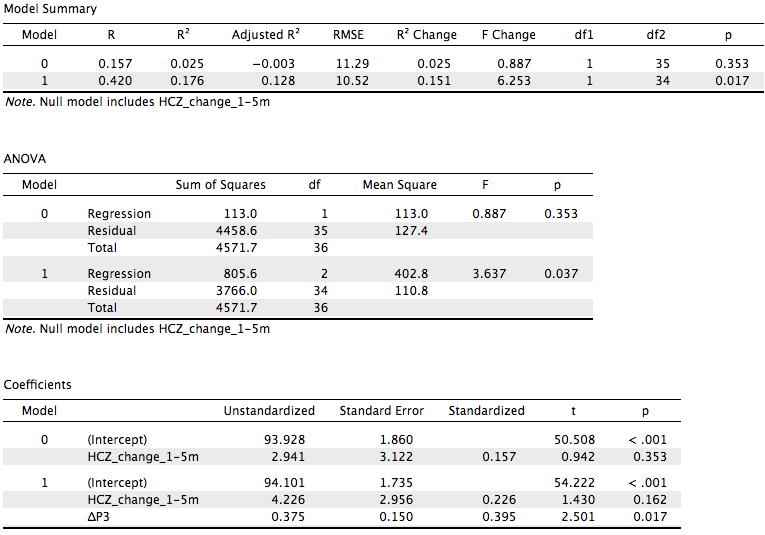


**Model 2. Head-circumference-z-score change between 1 and 5 months (HCZchange) and deltaP3 to predict MSEL scores at 5 months. Gambia only.**


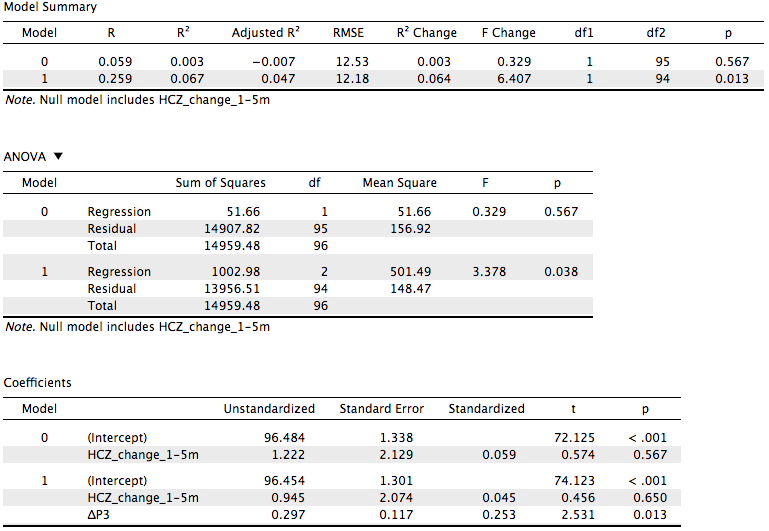

Supplement: Multimedia component 1 [file mmc1.docx]
